# Rational drug-design approach supported with thermodynamic studies — a peptide leader for the efficient bi-substrate inhibitor of protein kinase CK2

**DOI:** 10.1038/s41598-019-47404-0

**Published:** 2019-07-29

**Authors:** Maria Winiewska-Szajewska, Dawid Płonka, Igor Zhukov, Jarosław Poznański

**Affiliations:** 1 Institute of Biochemistry and Biophysics, Polish Academy of Sciences, Pawinskiego 5A, 02-106 Warsaw, Poland; 2 Department of Biophysics, Institute of Experimental Physics, Faculty of Physics, University of Warsaw, Pasteura 5, 02-093 Warsaw, Poland

**Keywords:** Thermodynamics, Peptides

## Abstract

Numerous inhibitors of protein kinases act on the basis of competition, targeting the ATP binding site. In this work, we present a procedure of rational design of a bi-substrate inhibitor, complemented with biophysical assays. The inhibitors of this type are commonly engineered by combining ligands carrying an ATP-like part with a peptide or peptide-mimicking fragment that determines specificity. Approach presented in this paper led to generation of a specific system for independent screening for efficient ligands and peptides, by means of thermodynamic measurements, that assessed the ability of the identified ligand and peptide to combine into a bi-substrate inhibitor. The catalytic subunit of human protein kinase CK2 was used as the model target. Peptide sequence was optimized using peptide libraries [KGDE]-[DE]-[ST]-[DE]_3–4_-NH_2,_ originated from the consensus CK2 sequence. We identified KESEEE-NH_2_ peptide as the most promising one, whose binding affinity is substantially higher than that of the reference RRRDDDSDDD peptide. We assessed its potency to form an efficient bi-substrate inhibitor using tetrabromobenzotriazole (TBBt) as the model ATP-competitive inhibitor. The formation of ternary complex was monitored using Differential Scanning Fluorimetry (DSF), Microscale Thermophoresis (MST) and Isothermal Titration Calorimetry (ITC).

## Introduction

Protein kinases catalyze the transfer of the γ-phosphate from ATP to the acceptor group of a specific amino acid of a target protein^1^. Protein kinases are attractive targets in cancer treatment, since the overexpression or mutation of particular kinases has been associated with cancerogenesis^2–4^. The majority of available protein kinase inhibitors act on the basis of competition, targeting the ATP binding site. The structure of the latter is highly preserved in various protein kinases, and thus, in spite of their high affinity, the inhibitors of this type may not be selective enough for a particular protein kinase. However, in addition to the ATP-binding site, each protein kinase has a specific binding site for the target protein(s), which enables the recognition of a so-called consensus sequence, identifying residues that preferably flank the phosphorylation acceptor site. This sequence, whose presence is necessary and sufficient to be recognized by a particular kinase, describes the general target specificity of the protein kinase^5^. Short peptides or peptide-mimetics that block the substrate binding site are therefore another group of potentially selective inhibitors of protein kinases^6–8^. However, due to the nature of kinase interaction with a target protein, the ligands of such type, mimicking that interaction, are rather weak binders.

To overcome all those problems and to enhance both the affinity and the specificity, bi-substrate inhibitors have been designed^9,10^. Ligands of this type target simultaneously the ATP- and substrate-binding sites, therefore, they should be designed on the basis of the shape and the location of natural substrates^9^. Such inhibitors are usually engineered by coupling ligands targeting ATP-binding site with a peptide fragment that determines the specificity^11^. This concept has been implemented leading to some moderately efficient inhibitors, consisting of a peptide fragment conjugated with either adenosine^12–15^ or nonhydrolyzable ATP derivatives^16–18^. Alternatively, a peptide may be conjugated with an ATP-competitive inhibitor^11,19–22^. This concept of the protein kinase inhibitor design is very promising, since the combination of two substrates required by the target enzyme may display very high specificity^23^.

However, there are several pitfalls to avoid in the development of new bi-substrate inhibitors. First of all, the interaction with the target peptides may be associated with conformational changes occurring in the kinase upon ligand binding. This includes global domain movement or local changes in the glycine rich P-loop, C-helix and activation loop^6,24^. Since the ligand-induced conformational changes in the target protein are still difficult to predict, we decided to support molecular modeling with experimental thermodynamic studies to identify the preferred sequence of the peptide part, and to check experimentally whether possible conformational changes interfere with the binding of the ATP-competitive part of a potential bi-substrate inhibitor of CK2α.

CK2 is a pleiotropic kinase that regulates many independent processes in cells because of its ability to phosphorylate multiple proteins (until now over 200 substrates of this kinase have been identified)^25^. Unlike the majority of Ser/Thr protein kinases, which recognize basic phosphoacceptor sites, the consensus sequence of CK2 displays preference towards acidic residues flanking the phosphate donor. Minimal consensus requires the presence of glutamate, aspartate, or occasionally phosphoserine (Sp) or phosphotyrosine (Yp) at the position +3 relative to the phosphoacceptor site. Although a single acidic residue at this position is sufficient for recognition, the presence of multiple acidic amino acids located upstream is strongly preferred. The significance of individual residues at a given position depends on the overall peptide sequence. An acidic residue at position +1 is almost as important as that at +3, while acidic residue at +2 is rather important for peptides in which no other acidic residues are located upstream^26,27^. Summarizing, the general consensus sequence, most frequently recognized by CK2, is X-[ST]-X-X-[EDSpYp]-X, where X is preferably acidic^5,26–28^.

Although the contribution of protein kinase CK2 to neoplasia formation remains unclear, CK2 has become a therapeutic target in anticancer treatments^29^. As for the most protein kinases, the majority of CK2 inhibitors target the ATP-binding site. There are also several reports regarding very promising bi-substrate inhibitors against CK2^30–33^. Most of them were designed using a common general approach. Enkvist *et al*. developed a highly potent inhibitor ARC-1502 (K_i_ = 0.5 nM), a conjugate of 4,5,6,7-tetrabromo-1*H*-benzimidazole with peptides containing multiple aspartic acid residues^30^, and was very successful in further optimization^32^. More recently, a bi-substrate inhibitor K137-E4 (IC_50_ = 25 nM), also derived form tetrabromo-1*H*-benzimidazole, was reported by Cozza *et al*., who combined K137 inhibitor (N1-(4,5,6,7-tetrabromo-1H-benzimidazol-2-yl)-propane-1,3-diamine) with Glu_4_ tetrapeptide, highly improving both the affinity and selectivity towards CK2^33^. In both cases, the design of inhibitors originated from a low-mass competitive inhibitor, to which acidic residues were linked without any broader sequence optimization.

Designing bi-substrate inhibitors by the independent optimization of the part targeting ATP-binding site, and peptides or peptidomimetics targeting substrate binding site may be a good alternative. In the broader view, this approach may be useful for designing generally bivalent inhibitors (i.e. inhibitors targeting two different sites of a protein, not necessarily substrate binding sites^34^) as there are already known some non-classic CK2 peptide-derived inhibitors that target the interface between the catalytic domain (CK2α) and the regulatory one (CK2β), i.e. CAM7117 with K_d_ = 150 nM^35^. There are also some CK2β motifs known to interact with other binding partners of CK2^36^, so peptides or peptidomimetics interfering with these interactions may also be a promising alternative.

Herein, we propose a rational approach based on the independent screening for efficient ligands and peptides, both performed with thermodynamic methods, that score the ability of particular compounds to combine further into an efficient bi-substrate inhibitor. We present the first step of rational design of the bi-substrate inhibitor. We tested three libraries of peptides, originated from the known consensus sequence of CK2, to identify the optimal one with the highest affinity toward the catalytic subunit of human protein kinase CK2. We further verified the possibility of combining the developed peptides with an ATP-competitive ligand to form an efficient bi-substrate inhibitor, using tetrabromobenzotriazole (TBBt) as a model ATP-competitive inhibitor. Finally, we analyzed the inhibitory activity of an ad hoc synthesized bi-substrate ligand.

## Results and Discussion

### Peptide library screening against CK2

Peptides that efficiently bind to the target protein were selected using pull-down method with immobilized His-tagged catalytic subunit of human CK2 (CK2α), and further identified using liquid chromatography-mass spectrometry (LC-MS). Three peptide libraries were tested. The first one, [KGDE]-[DE]-[ST]-[DE]_3_-NH_2_, contained 128 hexapeptides originating directly from the CK2 consensus sequence. The next library, containing 256 heptapeptides, was divided into two separate sub-libraries: [KGDE]-[DE]-[S]-[DE]_4_-NH_2_ and [KGDE]-[DE]-[T]-[DE]_4_-NH_2_, each consisting of 128 peptides. This approach was applied to overcome the substantial difference in the efficiency of synthesis of threonine- and serine-containing peptides.

**Figure 1:**
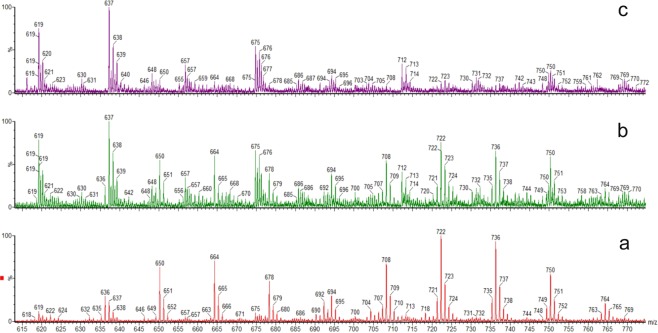
Mass Spectra of [KGDE]-[DE]-[ST]-[DE]_3_ -NH_2_ library pull-down experiment (**a**) library (**b**) eluent of the library from immobilized His-tagged CK2α column (**c**) eluent of the library from column without CK2α (control).

Signals identified in the MS spectra were assigned to a given peptide according to the indicated mass. However, this procedure led to several ambiguities, since a number of different peptides in the library displayed the same mass. LC-MS data was collected independently for the initial [KGDE]-[DE]-[ST]-[DE]_3_-NH_2_ library (Fig. 1a), and for the eluent released after passing this library through the column with immobilized hCK2α (Fig. 1b). Although in both runs the same peaks (*i*.*e*. M/z) were identified, their relative intensities visibly varied. We assumed that peptides displaying higher affinity to the protein should be enhanced in the second run. Some peptides were excluded from the further analysis due to their unspecific binding to the column’s resin (Fig. 1c). Finally, three peaks with substantially increased relative intensity in the eluent fraction were identified.

The first peak of 750 Da mass corresponds to an ensemble of six hexapeptides: EETEED-NH_2_, EETEDE-NH_2_, DETEEE-NH_2_, EETDEE-NH_2_, EDTEEE-NH_2_ and EESEEE-NH_2_, while the next two of 763 and 764 Da mass were identified as KETEEE-NH_2_ and EETEEE-NH_2_, respectively. The same procedure was performed for the two other libraries leading to the results consistent with those obtained for the first library (See Supplementary Figs S1, S2 and Table 1S).

Comparison of peptides identified in the pull-down assays pointed to favored peptides exhibiting two features: (1) glutamate residues flanking threonine/serine that undergo phosphorylation by CK2 (at most single aspartate residue) and (2) the N-terminal lysine. Nine hexapeptides (EETEEE-NH_2_; KETEEE-NH_2_; EESEEE-NH_2_; KESEEE-NH_2_; EETEED-NH_2_; EETEDE-NH_2_; DETEEE-NH_2_; EETDEE-NH_2_; EDTEEE-NH_2_) were synthesized in a larger-scale and subjected to thermodynamic studies. DDTDDD-NH_2_ peptide was also used to compare the advantage of glutamic acid over the aspartic acid. Binding affinity to hCK2α for all of the above peptides was assessed semi-quantitatively, using relaxation-filtered NMR spectroscopy.

Transverse relaxation rate (R_2_), which reflects the efficiency of the spin-spin exchange mechanism, depends on the hydrodynamic properties of the molecule, in particular on its rotational diffusion in solution. The experiment senses changes in ^1^H relaxation rates caused by the presence of hCK2α, binding to which decreases average rotational diffusion of the solute and thus almost uniformly affects relaxation rates of all ^1^H nuclei. In general, the transverse relaxation is much faster for larger molecules, whose nuclear spins experience much more efficient exchange^37,38^. Dynamic properties of the peptide, when virtually bound to the protein, resemble those of its molecular target. Assuming a fast exchange between free peptide and low-populated protein-bound state, the apparent properties of the free peptide are affected by the contribution of the bound form to the overall nuclear relaxation, which visibly increases the efficiency of spin-spin exchange. Assuming that properties of the protein target do not vary significantly upon ligand binding, the stronger the peptide binding to its molecular target, the faster the transverse relaxation is.

**Figure 2:**
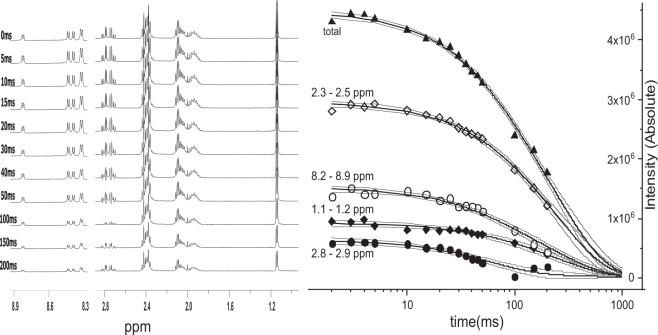
Relaxation-filtered NMR experiment for the EETEED-NH_2_ peptide in the presence of hCK2α. Ensemble of NMR spectra acquired with the increasing relaxation filter delay (**a**). Calculated intensity of different regions from NMR spectra in function of the relaxation filter delay (**b**).

To ensure that the observed effect was not associated with individual relaxation rates of free peptides in the solution, we performed a relaxation-filtered version of the experiment, with the relaxation delay varying in the range of 1 up to 200 ms. We observed substantial intensity variation of particular resonance lines caused by the increase of the delay, which were further quantitatively analyzed according to the model of two-exponential decay. The results of the analysis performed for the EETEED-NH_2_ peptide are shown in Fig. 2.

**Figure 3:**
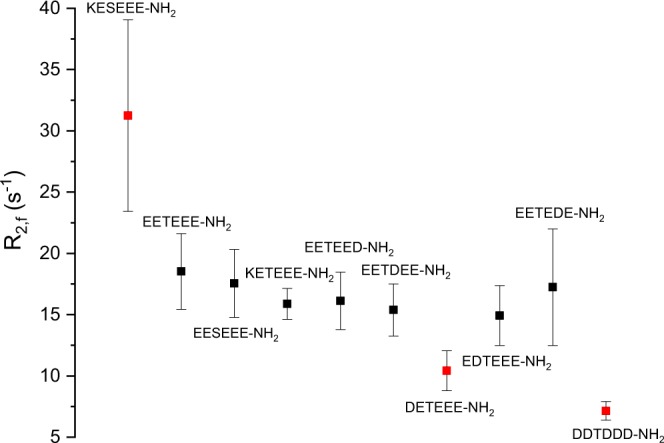
Transverse relaxation rates estimated for various protein-peptide complexes (R_2,f_),

For each peptide we globally analyzed signals determined for the set of the representative ^1^H resonances (see methods), assuming that the faster relaxation rate (R_2,f_) corresponds to the relaxation processes averaged over free and bound states, thus revealing the population of molecules interacting with the target protein. The slower one (R_2,s_) describes the relaxation of molecules that did not sense the protein. The higher R_2,f_ (or R_2,f_/R_2,s_ ratio), the higher population of the peptide is bound, implying its stronger binding to the protein. The estimated relaxation rates are summarized in Table 1 and shown in Fig. 3.

**Table 1:** Globally estimated relaxation rates for the free peptide (R_2,s_) and in the presence of hCK2α (R_2,f_).

Peptide	R_2,f_ (s^−1^)	R_2,s_ (s^−1^)
KESEEE-NH_2_	31.3 ± 7.8	2.5 ± 0.3
EETEEE-NH_2_	18.5 ± 3.1	3.1 ± 0.3
EESEEE-NH_2_	17.5 ± 2.8	2.3 ± 0.4
KETEEE-NH_2_	15.9 ± 1.3	2.6 ± 0.3
EETEED-NH_2_	16.1 ± 2.3	2.2 ± 0.5
EETDEE-NH_2_	15.4 ± 2.1	2.3 ± 0.5
DETEEE-NH_2_	10.4 ± 1.6	2.2 ± 0.6
EDTEEE-NH_2_	14.9 ± 2.5	1.5 ± 0.7
EETEDE-NH_2_	17.2 ± 4.8	3.7 ± 0.5
DDTDDD-NH_2_	7.1 ± 0.8	~1

All of the above data indicate that the KESEEE-NH_2_ peptide binds subtly stronger than any of the other tested peptides. Remaining peptides that do not carry the aspartic acid at position −2 relative to Ser/Thr bind to the hCK2α with comparable affinity, regardless the arrangement of further residues. Interestingly, Lys at position −2 improves the affinity for peptide with serine at the acceptor site, while no such effect is observed for peptide with threonine. This may suggest that some tiny differences between binding of serine and threonine substrates take place, therefore allowing CK2 to discriminate them. The observed difference in binding of KESEEE-NH_2_ and EESEEE-NH_2_ must result from electrostatic interactions rather than from steric boundaries, since DESEEE-NH_2_ binds to hCK2α with lower affinity. So, one may assume that the interaction pattern involving the first residue may depend on its type, and the preferred location of the N-terminal Glu may differ from that of Lys. This variation should be taken into account during the design of a linker to the low-mass ligand. Finally, DDTDDD-NH_2_, which corresponds to the C-terminal part of the commercially available reference peptide RRRDDDSDDD (used in the biochemical assays), binds visibly weaker to hCK2α.

### Determination of binding affinity

**Figure 4:**
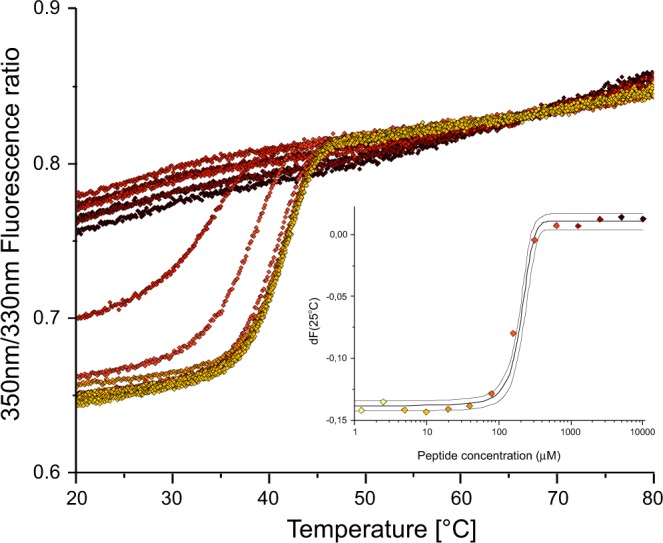
Thermal denaturation profile of KESEEE-NH_2_-hCK2α complex monitored as 350 nm/330 nm fluorescence ratio recorded at the increasing KESEEE-NH_2_ peptide concentration (from the lowest – yellow, to the highest – brown). For clarity, all curves were aligned at the high-temperature asymptotes. Nested graph summarizes changes in the fluorescence at 25 °C as a function of KESEEE-NH_2_ concentration.

All peptides that were identified as strong binders in NMR relaxation-filtering experiments (i.e. those that do not carry Asp in the sequence) were further studied semi-quantitatively, with low volume differential scanning fluorimetry (nanoDSF). The representative thermal unfolding profiles recorded for hCK2α in the presence of increasing concentration of KESEEE-NH_2_ are presented in Fig. 4.

Analogous experiments were performed for three other peptides (See Supplementary Fig. S3). All tested peptides destabilize the protein, and for all of them the decrease of the melting temperature was found dependent on the peptide concentration. For the high-temperature region (i.e. T > 65 °C in Fig. 4) a minor dependence on peptide concentration is indicative of its unspecific binding to the unfolded form of the protein, while at the lower temperature binding of peptides causes a strong decrease of 350 nm/330 nm fluorescence intensity ratio. These changes were used to estimate the binding affinity at 25 °C, assuming the single binding site model. The same procedure was applied to the data collected at 75 °C. Interestingly, dissociation constants estimated at 25 °C and at 75 °C differ visibly (K_d_ for KESEEE- NH_2_ at 75 °C is ~1 mM and 0.35 mM at 25 °C), therefore indicating that peptides bind to both folded and unfolded state of hCK2α. It could be hypothesized that the destabilization of the protein at low peptide concentration may be caused by unfavorable entropy balance upon binding of such hydrophilic ligands.

**Figure 5:**
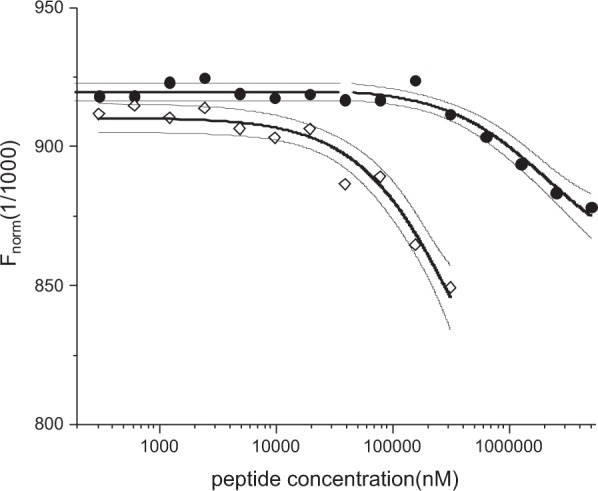
Microscale thermophoresis (MST) data for binding of: KESEEE-NH_2_ (diamonds) and commercially available peptide – RRRDDDSDDD (circles) to hCK2α.

The general order of peptides derived from nanoDSF is consistent with that obtained in the relaxation-filtered NMR experiments, confirming again that the binding affinity of KESEEE-NH_2_ is the highest (Table 2). The thermodynamic parameters of binding of KESEEE-NH_2_ peptide by hCK2α were also determined, with Microscale Thermophoresis, and further compared with the affinity of a commercially available reference peptide, RRRDDDSDDD (Table 2 and Fig. 5).

**Table 2:** nanoDSF-, MST- and ITC-derived thermodynamic parameters of binding of the peptides and TBBt to hCK2α, separately and for their ternary complexes.

titrate	nanoDSF				
	hCK2α				hCK2α + TBBt
titrant	KESEEE-NH_2_	EESEEE-NH_2_	KETEEE-NH_2_	EETEEE-NH_2_	KESEEE-NH_2_
Kd(mM)	0.35 ± 0.02	1.58 ± 0.06	0.95 ± 0.05	0.96 ± 0.03	0.45 ± 0.02
ΔG (kJ/mol)	−19.73 ± 0.14	−15.99 ± 0.09	−17.25 ± 0.13	−17.23 ± 0.08	−19.10 ± 0.11
**titrate**	**MST**				
	**hCK2α**		**hCK2α + KESEEE-NH**_**2**_		**hCK2α + TBBt**
**titrant**	**KESEEE-NH**_**2**_	**RRRDDDSDDD**	**TBBt**		**KESEEE-NH**_**2**_
Kd(mM)	0.39 ± 0.21	2.1 ± 0.7	(96 ± 44)∙10^−6^	(86 ± 28)∙10^−6^	0.36 ± 0.23
ΔG (kJ/mol)	−19.5 ± 1.3	−15.3 ± 0.8	−40.1 ± 1.1	−40.3 ± 0.8	−19.7 ± 1.6
**titrate**	**ITC**				
	**TBBt**		**TBBt**		
**titrant**	**hCK2α**		**hCK2α** + **KESEEE**		
K_d_(µM)	0.06 ± 0.03		0.10 ± 0.07		
ΔG (kJ/mol)	−41.3 ± 1.1		−40.0 ± 1.6		
ΔH (kJ/mol)	−25.1 ± 1.2		−17.7 ± 1.7		
ΔS (J/mol/K)	47 ± 8		70 ± 11

The peptide at concentration of approximately 1 mM causes hCK2a unfolding, which is manifested by the increase of fluorescence and distortion of raw MST data. This observation confirms the results obtained with nanoDSF, proving again that the peptide binds to the unfolded protein with K_d_ of about 1 mM. It should be emphasized that the binding affinity of KESEEE-NH_2_ towards hCK2α (K_d_ = 0.39 ± 0.21 mM) is significantly higher than that of commercially available RRRDDDSDDD peptide (2.1 ± 0.7 mM, ~1mM^39^).

### Testing the cross-dependency of ligand and peptide binding

**Figure 6:**
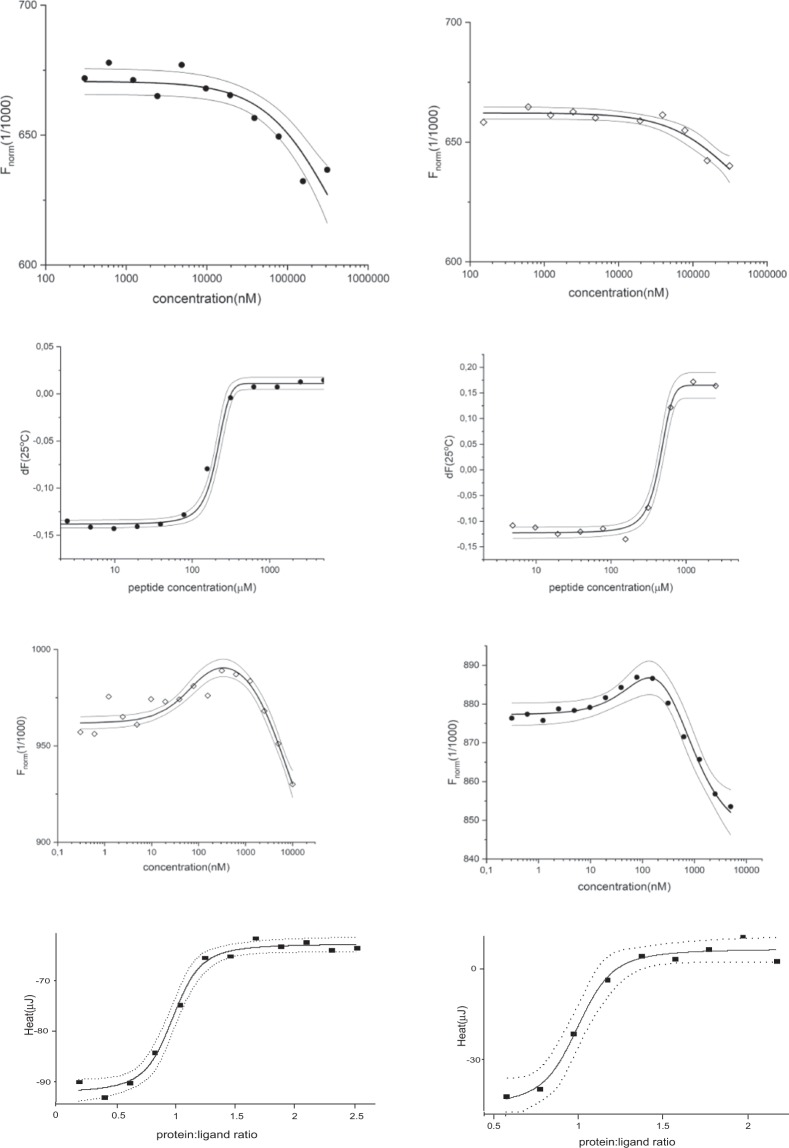
Ternary system analysis. MST data for binding of: (**a**) KESEEE-NH_2_ to hCK2α (left panel) and to TBBt/hCK2α complex (right panel) (**c**) tetrabromobenzotriazole to hCK2α (left panel) and KESEEE-NH_2_/hCK2α complex (right panel). (**b**) nanoDSF data for binding of KESEEE-NH_2_ to hCK2α (left panel) and to TBBt/hCK2α complex (right panel). (**d**) ITC data for TBBt binding to hCK2α (left panel) and to KESEEE-NH_2_/hCK2α complex (right panel).

To verify the ability of the identified KESEEE-NH_2_ peptide to bind with a low-mass ligand and form an efficient bi-substrate inhibitor, the interactions between the peptide, protein and TBBt (model ligand that binds at the ATP-binding site) were monitored using three methods - ITC, MST and nanoDSF. Combination of these techniques allowed for determination of the formal binding constant of peptide and ligand to the free protein, as well as peptide to ligand-protein complexes and ligand to peptide-protein complexes (Fig. 6 Table 2)

Comparison of peptide affinity towards the *apo* form of hCK2α and the hCK2α/TBBt complex was performed using nanoDSF and MST. Both methods confirmed that the presence of TBBt does not significantly affect peptide binding. The same applies to the KESEEE-NH_2_ interference with the TBBt binding, which effect was studied with ITC and MST. The corresponding values of dissociation constant remain the same within the experimental error. It could be thus concluded that the presence of peptide does not change the TBBt affinity, so both of them can be used as templates for designing a bi-substrate inhibitor.

### Molecular modeling of bi-substrate inhibitor

**Figure 7:**
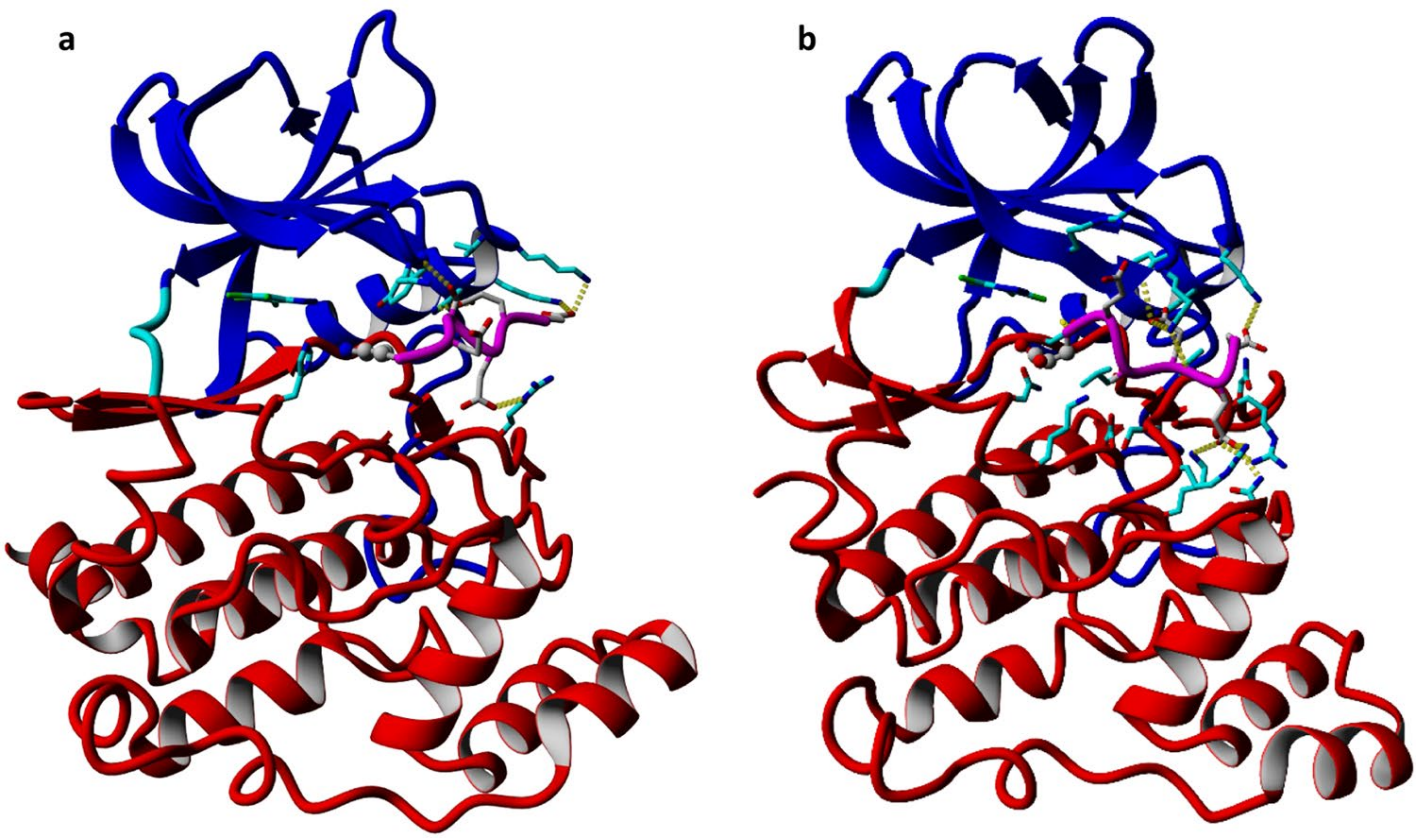
Snapshots of the Molecular Dynamic trajectory performed for the ternary complex of hCK2α and TBBt with KESEEE-NH_2_ (**a**) and with EESEEE-NH_2_ (**b**). The peptide backbone is denoted in magenta with the N-terminal Lys/Glu residue in ball-and-stick representation.

Molecular modeling of the ternary complex of hCK2α, TBBt, and EESEEE-NH_2_ or KESEEE-NH_2_ peptide was performed by a combination of modeling by homology with iterative modification of the ligand peptide followed by restrained molecular dynamics. The final structure of both complexes was found stable in terms of 30 ns unrestrained molecular dynamics (Fig. 7). The location of KESEEE-NH_2_ is stabilized by electrostatic interactions formed with proximal side-chains of Arg47, Lys49, Lys74, Lys76, Lys77, Lys158, His160, Arg191 and Lys198. All these interactions contribute to the stabilization of protein-peptide, which was estimated with FoldX to 4.2 kcal/mol. The *in silico* determined k_d_ = ~0.8 mM is therefore close to the value of 0.3 +/− 0.2 mM determined experimentally with MST. It is worth noting that the side-chain nitrogen of the N-terminal lysine of the peptide points towards TBBt, located at the ATP binding site, thus directing the way for setting up a bi-substrate ligand. The same procedure was applied for the EESEEE-NH_2_ peptide. In this case, the side-chain of the N-terminal residue was preferably oriented away from TBBt, therefore disqualifying side-chain of the N-terminal Glu as a potential linker, which could be however linked via the N-terminal amino group. Importantly, the complex with KESEEE-NH_2_ remained in the open conformation, while that with EESEEE-NH_2_ has switched to the closed one.

### Potency of bi-substrate inhibitor against human CK2α

To confirm the validity of our approach, we synthesized ad hoc a simple, bi-substrate compound, based on the optimized peptide sequence, that was conjugated by an amide bond formed between side chain of the N-terminal lysine and 7-COOH-Br_3_Bt. The inhibitory activity of this preliminary bi-substrate inhibitor, IC_50_ = 0.67 ± 0.15 µM, is comparable to that of TBBt (0.62 ± 0.28 µM), but higher than that of the leading 7-COOH-Br_3_Bt (8.0 ± 6.3 µM). Therefore, when compared with the affinity of the low-mass precursor, we obtained over 10-fold enhancement of inhibitory activity for bi-substrate ligand, while coupling of Glu_4_ with K137 improved the inhibitory activity only 5-fold^33^). This clearly exemplifies the potency of the proposed approach, proving the importance of the optimization of peptide sequence. However, taking into account IC_50_ values reported for CK2 bi-substrate inhibitors K137-E4 and ARC-1502 (25 nM^33^ and 2.7 nM^30^, respectively), it is clearly understandable that the low-mass ligand as well as the linker must be further optimized.

## Conclusions

In this work we presented a rationalized approach in CK2 drug design, in which the peptide part of a bi-substrate inhibitor was optimized to obtain an effective bi-substrate inhibitor. Combining experimental thermodynamic methods, we successfully screened three peptide libraries and identified the KESEEE-NH_2_ hexapeptide that binds to hCK2α with affinity higher than any of the studied peptides previously used as substrates for this kinase. We also proved that the binding of this peptide does not significantly attenuate the binding of an ATP-competitive ligand, which makes the proposed peptide a promising part of an efficient bi-substrate inhibitor. Molecular modeling additionally supports this hypothesis, clearly demonstrating that the linking of halogenated benzotriazole with the N-terminal lysine of the peptide may form an efficient bi-substrate ligand. In summary, the presented experimental approach supports the rational design of specific bi-substrate inhibitors of CK2, which also can be applied to other protein kinases.

## Methods

### Expression and purification of hCK2α

The catalytic subunit of human CK2, hCK2α, was expressed and purified according to the method described previously^40^.

### Peptide library synthesis

Three peptide libraries [KGDE]-[DE]-[ST]-[DE]_3_-NH_2_, [KGDE]-[DE]-[S]-[DE]_4_-NH_2_ and [KGDE]-[DE]-[T]-[DE]_4_-NH_2_ were synthesized by standard solid-phase peptide synthesis on TentaGel S RAM resin (Rapp polymere) using a Prelude peptide synthesizer (Protein technologies) with equimolar peptide building blocks. Side chain protecting groups were Boc, tBu and OtBu for Lys, Ser/Thr and Asp/Glu respectively. Cleavage was performed with 95:2.5:2.5% trifluoroacetic acid (TFA):triisopropylsilane:H_2_O, and the resulting peptides were precipitated with cold diethyl ether, centrifuged, the pellets were then dissolved in H_2_O and finally lyophilized. The compositions of libraries were analyzed by ESI-MS^41^. To avoid losing single peptides, the products were purified by dialysis against TBS buffer (25 mM Tris-HCl, 0.15 M NaCl, pH 7) and verified by ESI-MS using LC-MS separation on RP-C18 resin.

### Peptide synthesis

10 Peptides: EETEEE-NH_2_; KETEEE-NH_2_; EESEEE-NH_2_; KESEEE-NH_2_; EETEED-NH_2_; EETEDE-NH_2_; DETEEE-NH_2_; EETDEE-NH_2_; EDTEEE-NH_2_ and DDTDDD-NH_2_ were synthesized as described above. The products were purified by HPLC using Empower system (Waters) equipped with RP-amide semi-preparative column (5 µm particle size, 250 × 10 mm) (Ascentis). The mobile phase consisted of (A) 0.1% TFA in water and (B) 0.1% TFA with 90% acetonitrile in water. The purity and identity of lyophilized peptides was verified by ESI-MS using LC-MS separation. The final concentration was determined using Direct Detect Infrared Spectrometer (Merck).

### Bi-substrate inhibitor synthesis

4,5,6-tribromo-7-methyl-1H-benzotriazole (7-CH3-Br_3_Bt) (1) was obtained in good yield, according to the previously published procedure^42^. Subsequent oxidation with KMnO_4_ led to 7-COOH-Br_3_Bt (2) in 22% yield and high purity (see supplementary Fig. S4).

Bi-substrate inhibitor was synthesized and purified the same way as peptides described above, with 7-COOH-Br_3_Bt (2) as the first residue in sequence. The purity and identity of lyophilized compounds was verified by ESI-MS using LC-MS separation.

### Pull-down assays

Pull-down assays were used to identify peptides that strongly interact with CK2. For this purpose the Pierce™ His Protein Interaction Pull-Down Kit was used. Protein diluted in TBS buffer was incubated with cobalt resin supplemented in Kit (initially equilibrated with TBS buffer) at 4 °C for 1 h with continuous mixing. The resin was separated by centrifugation and the supernatant was removed. The resin was then washed five times with TBS buffer. Next, TBS buffer containing peptide library was added and incubated overnight at 4 °C with continuous mixing. To separate peptides that efficiently bind to the protein, the resin was centrifuged and washed two times with TBS buffer. Material bound to the column was eluted with 2 M glycine pH 2.5 and further identified by ESI-MS separation on RP-18 resin.

### Relaxation-filtered NMR spectroscopy

NMR samples containing tested peptides and hCk2α were prepared in 25 mM Tris–HCl (pH 7, 0.25 M NaCl) buffer with 10% D_2_O. The protein concentration was 25 μM, and a 100-fold peptide excess (2.5 mM) was used throughout the studies.

The scoring of the peptides’ binding affinities towards hCK2α was done according to the results of ^1^H transverse (T_2_) relaxation filtering experiment^43^. All spectra were recorded at 25 °C on Bruker Avance 500 NMR spectrometer (11.7 T) using 18 delays (0, 1, 2, 3, 4, 5, 10, 15, 20, 25, 30, 35, 40, 45, 50, 100, 150 and 200 ms). The spectra of 7500 Hz spectral width, 16k data points each, were collected using 16 scans with 2.0 s recycling delay preceded by 8 dummy scans.

NMR data were further processed and analyzed with the SpinWorks software (version 4.2.8.0), using cosine-squared window function and Gaussian filter resulting in 0.3 Hz broadening with no solvent filter applied prior to Fourier transformation. We analyzed ^1^H transverse relaxation rates of selected nuclei representative for each type of amino acid residues present in the tested peptides, i.e Hγ of Glu (2.3ppm), Hβ of Asp (2.8ppm), Hβ of Ser (3.8ppm), Hγ(CH_3_) of Thr (1.1 ppm) and Hε of Lys (2.9ppm), respectively. Additionally, the resonance signals of amide protons (8.2–8.9ppm) were also analyzed. We performed the relaxation-filtered experiment, with the relaxation delay varying in the range of 1 to 200 ms. Variation in the intensity of particular resonance signals were analyzed using the model of two-exponential decay (1):

1 $$y={A}_{1}\cdot \exp (t\,\ast \,{R}_{2,f})+A2\cdot \exp (t\,\ast \,{R}_{2,s})+{y}_{0}$$

where R_2,s_ and R_2,f_ are the transverse relaxation rates of the free and the fraction of the virtually bound peptide, respectively. For each peptide the global optimization procedure was applied for all identified resonance signals.

### Low-volume screening with differential scanning fluorimetry (nanoDSF)

The assay was carried out in 25 mM Tris–HCl (pH 7, 0.5 M NaCl) buffer with protein concentration preserved constant at 2.5 μM, while peptide concentrations varied in the 300 nM-10 mM range. The samples were loaded into nanoDSF Grade Standard Capillaries (NanoTemper Technologies), and analyzed using the Prometheus NT.48 nanoDSF device (NanoTemper Technologies). Thermal unfolding of the protein was monitored using a linear thermal ramp (1 °C/min; 20 °C to 80 °C) with an excitation power of 30%. Binding affinity of peptides was estimated from the ratio of 330 nm and 350 nm estimated at 25 °C and 75 °C for the native and unfolded forms of the protein, respectively. The data was analyzed using the numerical model implemented in Origin 9.0 package (www.originlab.com).

### Microscale Thermophoresis (MST)

The hCK2α sample was initially labeled with the commercially available NT-647 dye, using NanoTemper Protein Labeling Kit RED. The concentration of the ligand varied in the range of 100 nM-10 mM, while the concentration of the labeled protein was kept constant at 50 nM. All obtained data were analyzed according to the method described previously^44^, using appropriate model implemented in Origin 9.0 (www.originlab.com). Dissociation constants were estimated globally for a series of three independent pseudo-titration MST experiments, using effect of thermophoresis with temperature jump as the reporter of protein-ligand interaction.

### Isothermal titration calorimetry (ITC)

All ITC measurements were carried out at 25 °C with the Nano ITC calorimeter (TA Instruments), using 250 rpm stirring and 1000 s delay between succeeding injections to the sample cell of the volume 950 μl. The 12 injections of 20 μl volume following first pre-injection of a reduced volume (4 μl) were analyzed. The sample cell was initially filled with 4–10 μM TBBt solution, while 20–60 μM protein solution was placed in the syringe. Ternary complex formation was tested using the same experimental setup with the tested peptide present both in the cell and in the syringe at identical concentration of 500 μM. For each protein/ligand combination at least two titration experiments were done with different reagent concentrations. The data were further processed using the numerical model implemented in Origin 9.0 package (www.originlab.com), assuming existence of one type of binding sites of the apparent stoichiometry close to 1:1.

### Molecular modeling

Initial structure of the ternary complex of hCK2α with TBBt and KDS tripeptide was adopted from the accessible structures of TBBt bound to the catalytic subunit of maize CK2 (pdb1J91)^45^ and kinase Pim-1 with its substrate peptide (pdb3MA3)^22^. After replacement of the last three residues by a required sequence KES, the resulting structure was optimized and further relaxed with 5 ns molecular dynamics in the NPT ensemble, in the presence of explicit water molecules, with coordinates of protein backbone and serine of the ligand peptide constrained. In the successive steps, three Glu residues were added one-by-one at the C-terminus of the peptide, and each of these structures were successively subjected to 5 ns constrained MD, in which various distance restraints were tested to facilitate formation of salt-bridges with proximal basic residues of hCK2α. The final model of the ternary complex hCK2α/TBBt/KESEEE-NH_2_ was further subjected to 50 ns unrestrained MD performed in the NTP ensemble (298 K, 1 atm). The structure of the ternary complex hCK2α/TBBt/EESEEE-NH_2_ was adopted from that with KESEEE-NH_2_, and further subjected to 30 ns unrestrained molecular dynamics. All calculations were performed with Yasara Structure using Yasara2 force-field.

### *In vitro* inhibitory activity towards human CK2α

The ADP-Glo kinase assay (Promega) was used to measure the inhibition effect of the tested compounds. The kinase assay was carried in 96-well plate in volume of 25 μl in 20 mM Tris–HCl (pH 7.5) buffer, containing 100 ng hCK2α (1 μl), 10 μM CK2 substrate peptide RRRDDDSDDD (Biaffin GmbH & Co KG), 10 μM ATP, 20 mM MgCl_2_ and 2.5 μl of serially diluted ligand in DMSO. The reaction was initiated with enzyme in and kept going for 20 min at 30 °C. Reaction was stopped by adding 25 μl of ADP-Glo reagent. After incubation at room temperature for 40 min (to consume the remaining ADP) 50 μl of kinase detection reagent were added and incubated for additional 30 minutes to produce luminescence signal. Luminescence was measured with The SpectraMax iD3 Multi-Mode Microplate Reader (Molecular Devices). IC_50_ values were determined using three independent experiments, 7 inhibitor concentrations in the range of 1nM-1mM each, by global fitting of the sigmoidal dose-response equation implemented in Origin 9.0 package (www.originlab.com). Single experiment was analyzed for TBBt to confirm agreement with already published data^42,46,47^.

## Supplementary information

Supplementary

## Data Availability

The datasets generated during and/or analyzed during the current study are available from the corresponding author on a reasonable request.
