# Supplementary material for: Rational drug-design approach supported with thermodynamic studies — a peptide leader for the efficient bi-substrate inhibitor of protein kinase CK2
Source: Sci Rep. 2019 Jul 29;9:11018. doi: 10.1038/s41598-019-47404-0 (PMC6662822; doi:10.1038/s41598-019-47404-0)
Supplement: Supplementary file 1 — Supplementary [file 41598_2019_47404_MOESM1_ESM.pdf]

Rational drug-design approach supported with thermodynamic studies — a peptide leader for the efficient bi-substrate inhibitor of protein kinase CK2.

Maria Winiewska-Szajewska[1,2*], Dawid Płonka[1], Igor Zhukov[1], Jarosław Poznański[1]

[1] Institute of Biochemistry and Biophysics, Polish Academy of Sciences, Pawinskiego 5, 02-106 Warsaw, Poland

[2] Department of Biophysics, Institute of Experimental Physics, University of Warsaw, Ludwika Pasteura 5, 02-093 Warsaw, Poland

*corresponding author: Maria.Winiewska@fuw.edu.pl

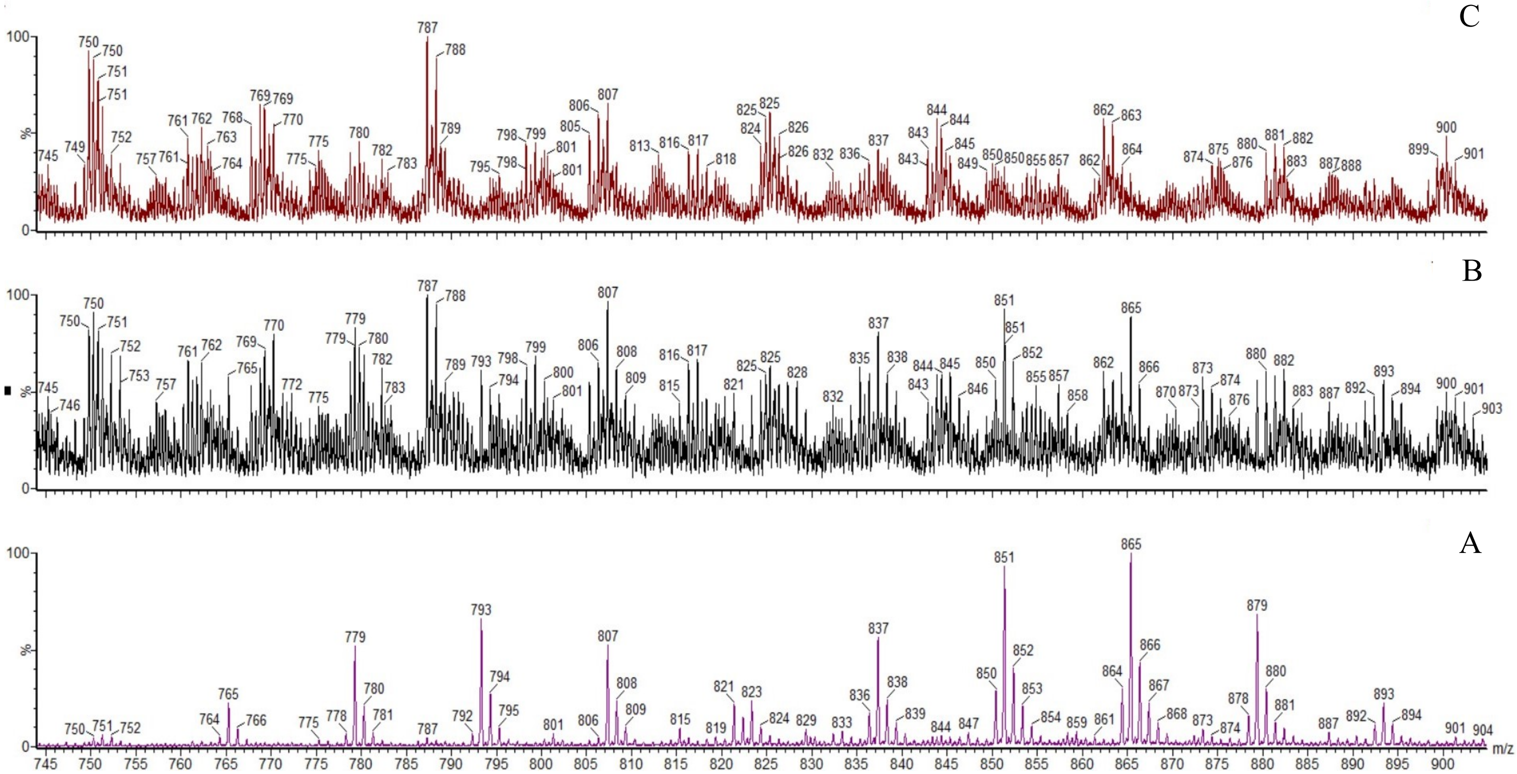

Fig. S1 MS Spectra of [KGDE]-[DE]-[T]-[DE]$_4$ library pull-down experiment A) solution of library B) eluent of library from immobilized His-tagged CK2α column C) eluent of library from column without CK2α (control).

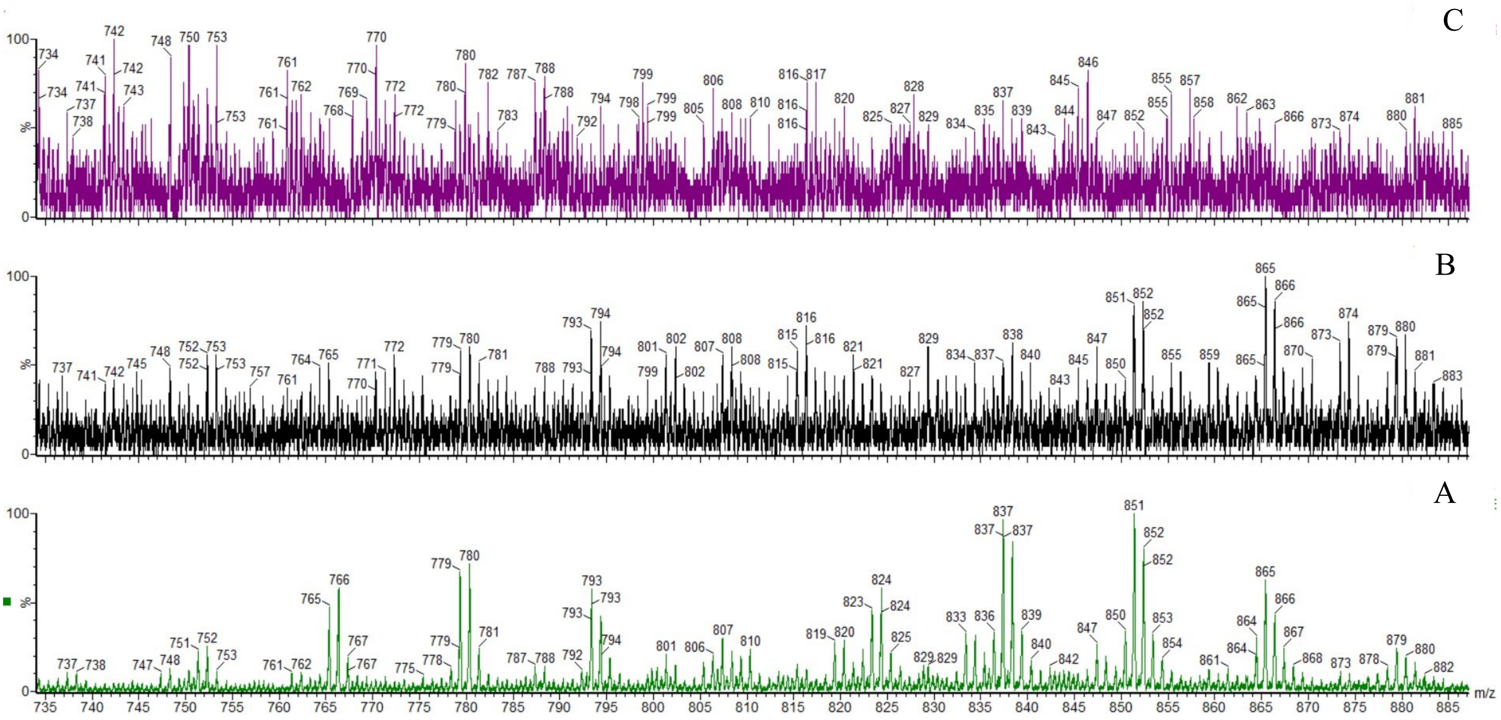

Fig. S2 MS Spectra of [KGDE]-[DE]-[S]-[DE]$_4$ library pull-down experiment A) solution of library B) eluent of library from immobilized His-tagged CK2α column C) eluent of library from column without CK2α (control).

Table 1S Signals identified in MS spectra (M/z) and peptides assigned according to the mass.

M/z	Peptide sequence
**library 2: [KGDE]-[DE]-[S]-[DE]$_4$**	
793	GDSEEEE-NH2
	GESEDEE-NH2
	GESEEED-NH2
	GESEEDE-NH2
	GESDEEE-NH2
865	EESEEDE-NH2
	EESEDEE-NH2
	DESEEEE-NH2
	EESDEEE-NH2
	EESEEED-NH2
	EDSEEEE-NH2
878	KESEEEE-NH2
879	EESEEEE-NH2
**library 3: [KGDE]-[DE]-[T]-[DE]$_4$**	
892	KETEEEE-NH_2
893	EETEEEE-NH_2

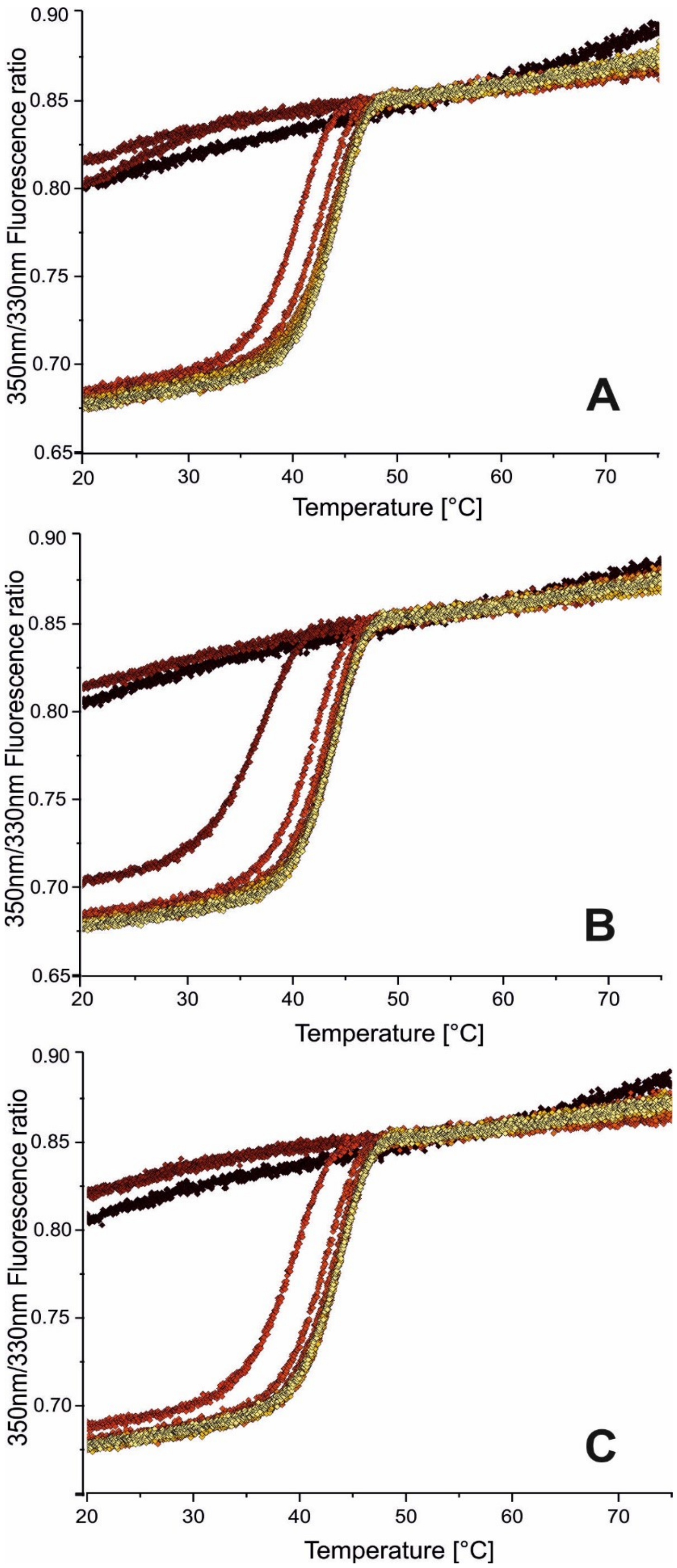

Fig. S3 Thermal denaturation monitored with the aid of 350nm/330nm fluorescence ratio recorded at increasing peptide concentration (from lowest – yellow color, to highest brown color). A) EETEEE-hCK2α complex B) EESEEE-hCK2α complex C) KETEEE-hCK2α complex. For presentation clarity all curves were superposed at high-temperature asymptotes.

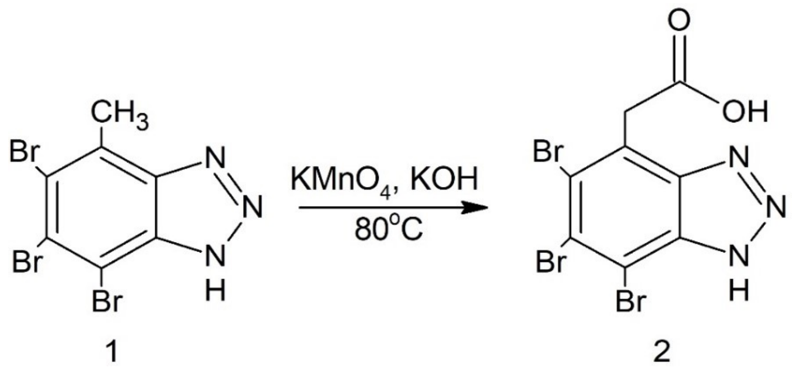

Fig. S4 Synthesis scheme for 4,5,6-tribromobenzotriazole-7-carboxylic acid (7-COOH-Br_3Bt -2).
